# A Transnosographic Self-Assessment of Social Cognitive Impairments (ACSO): First Data

**DOI:** 10.3389/fpsyt.2019.00847

**Published:** 2019-11-21

**Authors:** Jérôme Graux, Alix Thillay, Vivien Morlec, Pierre-Yves Sarron, Sylvie Roux, Baptiste Gaudelus, Zelda Prost, Lindsay Brénugat-Herné, Isabelle Amado, Shasha Morel-Kohlmeyer, Emmanuelle Houy-Durand, Nicolas Franck, Isabelle Carteau-Martin, Charlotte Danset-Alexandre, Elodie Peyroux

**Affiliations:** ^1^Pôle de Psychiatrie et d’Addictologie, CHRU de Tours, Tours, France; ^2^UMR 1253, iBrain, Université de Tours, Inserm, Tours, France; ^3^Reference Center for Cognitive Remediation and Psychosocial Rehabilitation (SUR-CL3R), Le Vinatier Hospital, Lyon, France; ^4^Reference Center for Cognitive Remediation and Psychosocial Rehabilitation, INSERM U894, Hôpital Sainte Anne, Paris, France; ^5^Resource Center for Cognitive Remediation and Psychosocial Rehabilitation of Lyon (CRR), UMR 5229, CNRS & Université Lyon 1, Université de Lyon, Lyon, France

**Keywords:** social cognition, self-assessment scale, schizophrenia, autism, bipolar, neurocognitive insight

## Abstract

Social cognition refers to the mental operations underlying social interactions. Given the major role of social cognitive deficits in the disability associated with severe psychiatric disorders, they therefore constitute a crucial therapeutic target. However, no easily understandable and transnosographic self-assessment scale evaluating the perceived difficulties is available. This study aimed to analyze the psychometric qualities of a new self-administered questionnaire (ACSo) assessing subjective complaints in different domains of social cognition from 89 patients with schizophrenia, schizoaffective disorders, bipolar disorders or autism. The results revealed satisfactory internal validity and test-retest properties allowing the computation of a total score along with four sub scores (attributional biases, social perception and knowledge, emotional perception and theory of mind). Moreover, the ACSo total score was correlated with other subjective assessments traditionally used in cognitive remediation practice but not with objective neuropsychological assessments of social cognition. In summary, the ACSo is of interest to complete the objective evaluation of social cognition processes with a subjective assessment adapted to people with serious mental illness or autism spectrum disorder.

## Introduction

Social cognitive processes refer to the mental operations underlying social interactions. These processes are frequently altered in patients with serious mental illnesses such as schizophrenia (1, 2), bipolar disorder (3), depression (4), borderline personality disorder (5), or autism spectrum disorder (6, 7) which have a significant impact on their daily lives. Indeed, alterations of social cognitive processes seem to predict quality of life (8–10), ability of functioning in the community (11–13) and, to a certain extent, occupational integration as well as relapses (9, 14–17). Given the major role of social cognitive deficits in serious mental illnesses, they constitute a crucial therapeutic target. Nevertheless, social cognition is not a unitary component (18), it encompasses several cognitive processes more or less independent from one another, with links between them leading to controversial debates. In 2013, the report of the SCOPE study (Social Cognition Psychometric Evaluation), mentioned that "there is no consensus on exactly which abilities define the construct" and that a "considerable conceptual and measurement-related overlap exists among domains". However, a consensual definition of this cognitive domain, and of the components of social cognition alterations that exist in psychiatry and mainly in schizophrenia emerged (19), encompassing: (1) emotional processes (EP), i.e. the ability to infer emotional information from others' facial expressions and vocal inflections; (2) theory of mind (ToM), i.e. the ability to represent others' mental states and to use these representations flexibly to understand, predict, and judge their behaviors; (3) attributional biases (AB): i.e. how individuals explain the causes of positive and negative events happening to them; and (4) social perception and knowledge (SP) i.e. the ability to decode and to interpret social cues (including social context processing and social knowledge).

Objective assessment of each of these dimensions is possible, even if psychometric quality of measures can be low (19). In addition, several batteries of tests are under development in order both to exhaustively characterize social cognitive disorders of psychiatric populations, and to standardize practices. However, the collection of subjective complaints in social cognition only relies on the clinical interview, without a specific questionnaire with the exception of the Observable Social Cognition Rating Scale (OSCARS; 20). Initially, this scale was developed to be an interview-based assessment specifically designed for individuals with schizophrenia. Yet, an easily understandable, transnosographic self-assessment scale should be useful to drive clinician's choice of therapeutic targets (21). In addition, self-assessment allows the person to be aware of his/her symptoms, his/her cognitive functioning and to discuss with the therapist about the negative consequences in daily life. It facilitates the therapeutic alliance and increases the patient's motivation for care, particularly in the field of cognitive remediation (22).

This study evaluates the psychometric qualities of a new French self-administered questionnaire (ACSo) assessing subjective complaints in social cognition. We selected heterogeneous groups of patients with schizophrenia, autism spectrum disorder, or bipolar disorder in order to promote a transnosographical approach and to provide psychometric information concerning the disabilities caused by these troubles.

## Material and Methods

### Participants

Eighty-nine patients: 43 individuals with schizophrenia (SZ), four with schizoaffective disorders, regrouped in a "schizophrenic spectrum" group, 24 with bipolar disorders, and 18 with autism spectrum disorder (ASD) without intellectual disability (DSM-5) were recruited from three psychosocial rehabilitation centers located in Tours, Paris, and Lyon (see **Table 1**). All participants were adults, with a minimum secondary school level, and clinically stabilized as confirmed by PANSS, BDI or YMRS scores.

**Table 1:** Participants' characteristics (mean ± standard deviation; min-max).

	Schizophrenic spectrum (N=47)	Bipolar disorders (N=24)	Autism Spectrum Disorder (N=18)	All patients (N=89)	Healthy controls (N=102)
	Mean ± SD (min-max)	Mean ± SD (min-max)	Mean ± SD (min-max)	Mean ± SD (min-max)	Mean ± SD (min-max)
**Age (years)**	33.9 ± 9.5 (18-53)	44.5 ± 11.7 (20-68)	26.5 ± 5.8 (18-37)	35.3 ± 11.4 (18-68)	24.1 ± 5.7 (18-45)
**Gender (F:M)**	12:30	13:11	4:17	29:58	54:48
**Education (years)**	11.9 ± 2.0 (9-17)	14.0 ± 2.7 (9-17)	12.4 ± 2.3 (9-15)	12.6 ± 2.4 (9-17)	13.5 ± 1.6 (9-17)
**PANSS total**	67.5 ± 13.6 (34-95)				
**PANSS positive subscore**	15.2 ± 4.4 (9-26)				
**PANSS negative subscore**	19.8 ± 5.7 (8-32)				
**BDI**		8.9 ± 6.4 (0-23)			
**YMRS**		0.2 ± 0.6 (0-2)			
**ADOS social and communication score**			13.6 ± 3.7 (9-19)		
**ADOS restricted, repetitive behavior**			2.3 ± 1.8 (0-6)		
**Overall intellectual quotient**			100 ± 12 (80-122)		
**Verbal quotient**			112 ± 16 (83-139)		
**Performance quotient**			100 ± 17 (80-136)		

Symptoms were assessed (1) in schizophrenia with the Positive and Negative Syndrome Scale for Schizophrenia (PANSS; 23, 24) and the Self-evaluation of Negative Symptoms (SNS; 25, 26), (2) in bipolar disorders with the Beck Depression Inventory (BDI; 27, 28) and Young Mania Rating Scale (YMRS; 29, 30), and (3) in autism with the Autism Diagnostic Observation Schedule-Generic (ADOS-G; 31).

Healthy control participants (HC; n = 102) were enrolled from the Le Vinatier Hospital in Lyon (n = 73) and from the University Hospital of Tours (n = 29). None of the HC had a previous history of neuropsychiatric troubles according to the MINI (30, 32) and were not taking any psychotropics, nor did they have any addictive disorder.

The study was carried out in accordance with the Declaration of Helsinki and was approved by the local ethical committee (CPP Lyon Sud Est IV, No. 15/041; ANSM, No. 2015-A00580-49). Written informed consent was obtained from all participants. Control participants were paid 30 Euros for their participation.

### Self-Assessment of Social Cognition Impairments (ACSo)

The scale was developed within the framework of a partnership work allowed by the GDR 3557 carried by the Institute of Psychiatry. The tool was developed in two stages.

1. Discussions with experts in the field of neuropsychology in psychiatry and based on an extensive literature review allowed the formulation of different proposals specifically targeting each process involved in social cognition. Each item was then reformulated and simplified in order to be accessible to a maximum of patients, particularly those with severely disabling cognitive disorders, low levels of education, or intellectual disabilities.
2. A "consensual core" of items was selected using the "expert panel" method involving neuropsychologists, nurses, psychiatrists, and researchers working in several units specialized in cognitive remediation and rehabilitation.

Finally, a preliminary study was conducted on a first self-administered questionnaire comprising 20 items organized into four groups (emotional processes, social perception, and knowledge, ToM and attributional biases) of five items. The study of the psychometric qualities of this first scale (personal data) resulted in the proposition of a revised and shortened version with 12 items organized into four groups of three items. Each item is rated by the participant on a 5-point Likert scale from 0 "never" to 4 "very often."

### Clinical and Social Cognitive Assessments

Patients completed the ACSo but also specific self-administered assessments traditionally used in cognitive remediation practices. All patients completed The Subjective Scale To Investigate Cognition in Schizophrenia (STICSS; 33). The STICSS is a self-administered 21-item Likert-type scale exploring cognitive complaints in several domains that are commonly disturbed in schizophrenia (memory, attention, language, praxia). Empathy was assessed with the Questionnaire of Cognitive and Affective Empathy (QCAE; 34, 35) in SZ and with the Empathy quotient (EQ; 36, 37) in ASD. The QCAE is a self-administered 31-item scale assessing both cognitive and affective components of empathy. The EQ is a self-report questionnaire designed to measure empathy. It contains 60 items: 40 empathy items and 20 filler/control items.

Two components of social cognition were also assessed in SZ or ASD thanks to objective measures. ToM abilities were evaluated by the Movie for the Assessment of Social Cognition test (MASC; 38, 39). This movie features four characters who meet in a party. The video is paused at the end of each sequence and questions concerning the characters' mental states – emotions or feelings, thoughts and intentions – are proposed. Participants have to choose between four responses: the correct answer ("ToM"); an "under-mentalization" answer ("less ToM"); a literal answer, with no mentalization ("no ToM"); or an over interpretative response ("excessive ToM"). The total score is the sum of all "ToM" responses. To measure emotion recognitions, the TREF (Facial Emotion Recognition Test, 40) was selected. It assesses the ability to recognize six basic and universal emotions (joy, anger, sadness, fear, disgust, and contempt). The test includes 54 color photographs of six different models (three men and three women of different ages). Each emotion is presented with nine levels of intensity from 20 to 100% and is presented for 10 seconds, followed by an instruction to name the emotion expressed, using a forced choice among the six possible responses, providing an overall percentage of correct emotion recognition (global score).

### Data Analyses

Statistical analyses were performed using STATISTICA v10 software (Stat Soft, Inc.). The ACSo internal validity was first studied using Principal Component Analysis (PCA) in order to explore the data structure and to identify the underlying dimensions of the scale. The number of factors was defined by retaining only the eigenvalues > 1, located above the slope break observed on the eigenvalue plot (screen test criterion). If a 2 or more-factor solution was retained, factor analysis with Varimax rotation was then performed. Items were included in the factor structure if they loaded |0.40| or higher on a factor, and if the loading was at least |0.10| higher than the loading on any other factor. The Cronbach α coefficient was used to assess the internal consistency of each retained dimension. The accuracy of the measurement during the test-retest was studied by calculating the Intraclass Correlation Coefficient (ICC) on each retained dimension. The convergent and divergent validities of the scale were studied, using Pearson's product moment correlations by relating each dimension (sum of the items making up each factor) with the total scores of the SSTICS, the MASC, the TREF, the QCAE, and the EQ. ROC analysis was also performed to examine the ability of the ACSo score to discriminate the patients from the 102 HCs and thus to evaluate the sensitivity and the specificity of the scale.

## Results

Scores obtained from the ACSo and from both clinical and neuropsychological assessments in all patient groups and healthy controls are given in **Table 2**.

**Table 2:** Scores from the ACSo, the TREF, the MASC, the SSTICS, the QCAE and the EQ in all participants (mean ± standard deviation; min-max).

	Schizophrenic spectrum (N=47)	Bipolar disorders (N=24)	Autism Spectrum Disorder (N=18)	All patients (N=89)	Healthy controls (N=102)
	Mean ± SD (min-max)	Mean ± SD (min-max)	Mean ± SD (min-max)	Mean ± SD (min-max)	Mean ± SD (min-max)
**Self-assessment of social cognition impairments (ACSo)**					
Total score	17.3 ± 7.3 (2-31)	17.3 ± 6.9 (5-34)	22.2 ± 8.7 (8-36)	18.3 ± 7.7 (2-36)	9.9 ± 5.2 (0-28)
Emotional perception	4.0 ± 2.2 (0-8)	4.5 ± 1.8 (0-8)	5.0 ± 2.4 (0-9)	4.3 ± 2.2 (0-9)	2.1 ± 1.5 (0-7)
Social perception and knowledge	4.2 ± 2.6 (0-9)	4.1 ± 2.0 (1-8)	6.1 ± 2.7 (2-11)	4.5 ± 2.6 (0-11)	3.0 ± 1.9 (0-10)
Theory of mind	5.4 ± 2.4 (2-12)	4.2 ± 2.1 (1-8)	6.2 ± 2.9 (1-12)	5.2 ± 2.5 (1-12)	2.9 ± 1.7 (0-8)
Attributional style	3.8 ± 2.6 (0-9)	4.6 ± 3.4 (0-12)	4.9 ± 2.6 (2-10)	4.2 ± 2.9 (0-12)	1.9 ± 1.7 (0-8)
**Facial emotion recognition (TREF)**					
% of correct recognition	64.4 ± 10.1 (40.7-83.3)		61.5 ± 10.7 (40.7-76.0)	63.5 ± 10.3 (40.7-83.3)	73.2 ± 7.6 (50.0-94.44)
**Theory of Mind (Masc)**					
Total score	24.9 ± 4.0 (17-32)		26.3 ± 5.4 (15-32)	25.4 ± 4.0 (15-32)	32.7 ± 3.4 (21-40)
**Subjective Scale To Investigate Cognition in Schizophrenia (SSTICS)**					
Total score	31.2 ± 13.1 (5-70)	30.0 ± 15.4 (7-70)	31.1 ± 11.6 (15-58)	31.2 ± 13.1 (5-70)	
**Questionnaire of Cognitive and Affective Empathy (QCAE)**					
Cognitive empathy	52.7 ± 6.9 (39-68)				55.1 ± 8.6 (26-76)
Affective empathy	32.6 ± 3.4 (22-38)				34.8 ± 5.2 (22-48)
**Empathy Quotient (EQ)**					
Total score			27.7 ± 11.5 (8-52)		41.2 ± 10.4 (15-78)

**Figure 1:**
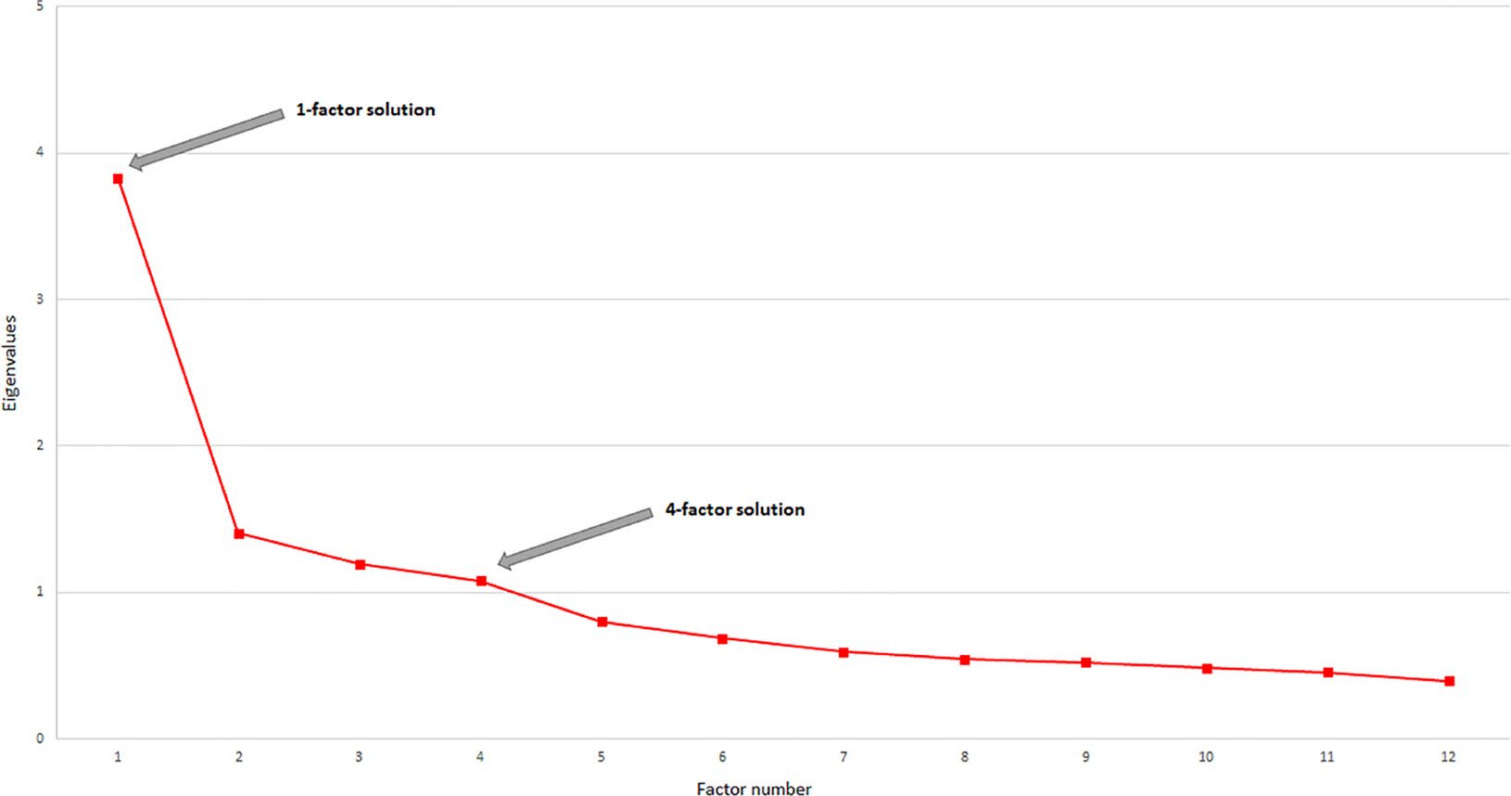
Scree plot of the eigenvalues for the 12 items of the ACSo. The two breaks in the slope after the first and the fourth eigenvalue are indicated by the arrows.

PCA performed on the 89 patients and the 12 items of the ACSo produced an eigenvalue plot with two breaks in the slope after the first and the fourth eigenvalue (**Figure 1**). Both a 1-factor and a 4-factor solution have therefore been retained.

### The 1-Factor Solution

For the 1-factor solution, the main dimension accounted for 34.4% of the total variance and all 12 factor loadings were > |0.40|. This structure was confirmed by the analysis of internal consistency; a Cronbach alpha coefficient of 0.822 demonstrated a good level of correlation between the 12 items, which was the highest alpha-if-item-deleted score (between 0.794 and 0.815). These results allowed to compute the ACSo total score (ATS) as the sum of the 12 items.

The test-retest reliability of the ACSo score was performed on a subset of 19 SZ who were asked to rate the scale twice over a period of 2 months. The ICC value obtained revealed a good test-retest reliability (ICC value = 0.828).

The ATS was not correlated to age (r = 0.070, p = 0.52, n = 89). In the entire patient population, the ATS was positively correlated with the SSTICS total score (cognitive complaints; r = 0.441; p < .0001, n = 82), achieving concurrent validity. Moreover, no significant correlations were found between the ATS and neuropsychological assessments of facial emotion recognition and theory of mind (respectively, TREF, percentage of correct recognition, r = -0.133, p = 0.32, n = 59; MASC, total score, r = 0.040, p = 0.77, n = 53) in the entire patient population. In SZ patients, the ATS was positively correlated with the QCAE affective score (affective empathy; r = 0.444; p = 0.018, n = 28) but no significant correlation was found with the QCAE cognitive score (cognitive empathy; r = 0.216, p = 0.27, n = 28). The ATS was negatively correlated with the EQ total score (empathy; r = -0.672; p = .002, n = 18) in ASD patients. In summary, increased ATS was associated with increased subjective complaints about cognitive functioning and empathy.

ROC analysis performed on the ATS provided a best cut-off of 13 with a sensitivity of 0.750 and a specificity of 0.724. The Area Under the Curve (AUC) was 0.810 (SE = 0.033).

### The 4-Factor Solution

For the 4-factor solution, the four dimensions accounted for 64.0% of the total variance. The first dimension included three items (ACSo-F1; items 4, 7 and 12; 17.3%), the second three items (ACSo-F2; items 3, 8 and 9; 16.6%), the third two items (ACSo-F3; items 1 and 10; 15.3%), and the fourth three items (ACSo-F4; items 2, 6 and 11; 14.8%; **Table 3**). The Cronbach α coefficients showed moderate or good internal consistency for these four sub-scores (0.672, 0.647, 0.645, 0.675, respectively). The ICC values for the four sub-scores computed on the subset of 19 SZ were 0.761, 0.781, 0.738, and 0.625 respectively, and showed moderate or good test-retest reliability.

**Table 3:** Factor loadings of the 12 items of the ACSo. The four *a priori* components of the scale are: Emotional Processes (EP), Theory of Mind (ToM), Attributional Biases (AB) and Social Perception and knowledge (SP). Items were included in the factor structure if they loaded |0.40| or higher on a factor (written in bold), and if the loading was at least |0.10| higher than the loading on any other factor. Loadings are highlighted in green when they agree with the *a priori* structure or in blue when they belong to another factor.

	*a priori* factor	F1	F2	F3	F4
Item 1	EP	0.066	0.263	**0.728**	0.298
Item 2	ToM	0.204	0.067	0.133	**0.776**
Item 3	SP	0.128	**0.691**	0.272	0.043
Item 4	AB	**0.797**	0.068	0.198	0.220
Item 5	SP	**0.528**	**0.440**	**0.404**	0.040
Item 6	ToM	0.218	0.193	**0.442**	**0.608**
Item 7	AB	**0.775**	0.036	0.166	0.078
Item 8	EP	0.116	**0.761**	0.096	0.197
Item 9	SP	0.097	**0.711**	0.025	0.117
Item 10	EP	0.146	0.028	**0.838**	0.022
Item 11	ToM	0.078	0.139	0.000	**0.758**
Item 12	AB	**0.630**	0.324	-0.269	0.181

Interestingly, the four factors identified overlapped partially with the four *a priori* components of the scale. ACSo-F1 included items 4, 7, and 12 exploring the "attributional biases" component, ACSo-F2 included items 3 and 9 exploring the "social perception and knowledge" component, ACSo-F3 included items 1 and 10 exploring the "emotional processes" component and ACSo-F4 included the items 2, 6, and 11 exploring the "theory of mind" component. Item 5 was excluded from the four dimensions because factor loadings were divided between F1, F2, and F3 (with less than a |0.10| loading difference between factors). Item 8, designed to explore "emotional processes", was reassigned because of high factor loading on F2.

Considering correlations between objective and subjective assessments of social cognition components, no significant correlations were found between the ACSo-F3 and neuropsychological assessment of facial emotion recognition (TREF, percentage of correct recognition, r = -0.241, p = 0.066, n = 59), nor between ACSo-F4 and neuropsychological assessment of theory of mind (MASC, total score, r = -0.010, p = 0.965, n = 53) in the entire patients population.

## Discussion

To our knowledge, the self-assessment of social cognitive impairments (ACSo) is the first transnosographic scale that assesses subjective complaints of social cognitive impairments. ACSO revealed good psychometric properties allowing the description of relevant components of social cognition. The ASCo was designed for mental disorders associated with social cognitive impairments (schizophrenia, bipolar disorders, ASD, ADHD, depression or personality disorders). Its ease of use (few items, easily understandable, illustrated with concrete examples) constitutes a major asset for its application in severe psychiatric disorders with cognitive impairments like autism or schizophrenia.

Factor analysis performed on the 12 items of the ACSo allowed us to retain both a 1-factor and a 4-factor solution. Each solution provides understandings of the psychometric properties of the scale and is relevant in clinical practice. In fact, social cognition is not a homogeneous construct but includes several dimensions. Although these different dimensions are considered and assessed separately for both theoretical and practical reasons, they may not be independent from one another but rather partially overlapping (41).

The 1-factor solution has very good psychometric properties. All 12 items have very good internal consistency, suggesting that they all assess social cognitive complaints. This single dimension argues for a considerable measurement-related overlap between each components of social cognition, in agreement with the conclusions of the SCOPE study (19). Moreover, the high ICC confirms the good reliability of the scale. Overall, the ATS constitute a good assessment of subjective complaints of social cognition as a whole. Interestingly, the ATS was found to be correlated with all subjective assessments (EQ total score, QCAE affective empathy score and SSTICS total score) but not with objective neuropsychological assessments of facial emotion recognition and ToM (respectively, TREF total score and MASC total score). These results are consistent with recent studies focusing on "neurocognitive insight" in schizophrenia, i.e. awareness into cognitive deficits (42–48). These studies reported indeed that the lack of insight on psychiatric symptoms (49) could be extended to cognitive impairments. More precisely, in accordance with Potvin et al. (50), SZ patients have cognitive complaints but do not have a clear representation of the nature of their difficulties. While these studies focused on neurocognitive functioning, awareness into social cognition is only addressed in three studies (24, 51, 52). They also reported a lack of awareness of social functioning in SZ (24, 52). However, no objective assessments have been used in these studies to specifically measure alterations of social cognitive processes. Using objective and subjective assessments of social cognition, the present study strongly suggests an altered "social cognitive insight" in people with serious mental illness. Furthermore, the lack of correlation between the ATS and objective assessments may reflect an impaired insight of social cognitive functioning but may also result from our objective measures focusing only on two specific processes (i.e. facial emotion recognition and ToM). In fact, even if several research teams are currently working on the validation of social cognition batteries, to date there is no neuropsychological tool assessing social cognition as a whole.

A threshold value of 13 was identified for an optimal balance between sensitivity and specificity. In other words, this cut-off was found to discriminate optimally between HCs and patients. Social cognitive skills range from normal to pathological and constitute a transnosographical component of psychiatric diseases. Therefore, the cut-off does not constitute a diagnostic threshold but rather represents a significant level of social cognitive complaints compared to a healthy population.

Factor analysis also allowed us to consider a 4-factor solution. Interestingly, the four-factor identified overlapped partially with the four *a priori* components of the scale (attributional biases, social perception and knowledge, emotional perception, and ToM). Only one item was excluded from the sub-scores, which confirmed that the 4-factor solution seems to be very suitable for the data. This item, "I lack tact with others (for example, by giving my opinion, I may unintentionally hurt those around me)", could not be classified in one factor because factor loadings were divided between several factors. Therefore, this item belongs both to "attributional biases" and "social perception and knowledge" components. In fact, even if this item was intended to assess social perception complaints, it appears that it may also be the behavioral consequence of attributional biases. Thus, item 5 remains relevant for the calculation of the total score but should not be used in the calculation of the sub-scores. Even though item 8 was intended to assess emotional perception complaints, it has a high factor loading on the "social perception and knowledge" component and was therefore assigned to this dimension. This item ("I have trouble recognizing when someone is ashamed") designed to explore complaints about complex emotional perception actually implies a social context. Interestingly, Cronbach α coefficients from both "social perception and knowledge" and "emotional perception" are decreased when computed from the *a priori* items, suggesting a gain of internal consistency with the reassignment of both item 5 and 8 (F2: 0.639 < 0.647; F3: 0.591 < 0.645).

A limitation of this study is the relatively small sample size. Additionally, our sample is only representative of patients from psychosocial rehabilitation centers. Finally, if the lack of correlation between ATS and objective assessments of social cognition may be explained by an impaired social cognitive insight as we discussed earlier, we could not exclude the possibility of an uncertain convergent validity of the scale.

Directions for future research include characterizing the ACSo response profile for each psychiatric clinical category concerned with impaired social processes (schizophrenia, autism, bipolar disorders, borderline personality disorders, antisocial personality disorders, ADHD, depression etc.), in particular by comparing objective and subjective responses to determine the social cognitive insight of each category. Focusing more specifically on persons with schizophrenia, it could be interesting to study the possible overlaps between subjective complaints of social cognitive impairments assessed with the ACSo and other first-person approaches of social dysfunctions inspired by clinical phenomenology, which have recently been formalized in the form of psychometric scales (53–55) in order to investigate the relationships between their social cognition insight and their specific pre-reflexive consciousness of social world. Also, in agreement with the assumption of a continuum ranging from normal to pathological in social cognition alterations, a comprehensive study in a healthy population could provide information on the variability of social cognition complaints along with "social cognition insight" skills.

In summary, the ACSo is the first scale that allows the collection of complaints concerning social cognitive processes. The ACSo total score has good psychometric properties and thus provides a measure of the severity of subjective complaints in the field of social cognition. The four sub-scores from the 4-factor solution can be used by clinicians in order to identify the components of social cognition for which patients report the most complaints. The ACSo is of major interest to complete psychosocial rehabilitation assessments with a subjective evaluation of social cognition adapted to people with serious mental illnesses associated with cognitive disorders.

## Data Availability Statement

The datasets generated for this study are available on request to the corresponding author.

## Ethics Statement

The studies involving human participants were reviewed and approved by the local ethical committee (CPP Lyon Sud Est IV, No. 15/041; ANSM, No. 2015-A00580-49). The patients/participants provided their written informed consent to participate in this study.

## Author Contributions

JG, VM, P-YS, BG, ZP, LB-H, IA, EH-D, IC-M, CD-A, and EP made substantial contributions to conception and design. JG, AT, ZP, LB-H, SM-K, EH-D, IC-M, CD-A, and EP made substantial contributions to acquisition of data. JG, AT, SR, and NF made substantial contributions to analysis and interpretation of data. JG, AT, VM, P-YS, SR, BG, ZP, LB-H, IA, EH-D, NF, IC-M, CD-A, and EP participated in drafting the article. All authors contributed to, have approved the final manuscript and agree to be accountable for the content of the work.

## Funding

This research was supported by the Groupe de Recherche en Psychiatrie (GDR) 3557 – Institut de Psychiatrie and by Le Vinatier Hospital and Lyon 2 University [CSLV07].

## Conflict of Interest

The authors declare that the research was conducted in the absence of any commercial or financial relationships that could be construed as a potential conflict of interest.
